# Users' Experiences During Cardiac Recovery Following Acute Myocardial Infarction: A Qualitative Study With a Gender Perspective

**DOI:** 10.1002/nop2.70886

**Published:** 2026-10-16

**Authors:** Susana Santos‐Ruiz, Carmen Jerez‐Molina, Rocio Rodriguez‐Romero, Juan José Zamora‐Sánchez, Luis González‐De Paz

**Affiliations:** ^1^ Parc Taulí Hospital Universitari, Institut de Investigació i Innovació Parc Tauli (I3PT‐Cerca) Universitat Autònoma de Barcelona Sabadell Spain; ^2^ Campus Docent Sant Joan de Déu, Universitat de Vic – Universitat Central de Catalunya (UVic‐UCC) Grup d'Investigació DAFNiS (Dolor, Activitat Física, Nutrició i Salut) Sant Boi de Llobregat Spain; ^3^ Consorci d'Atenció Primària de Salut Barcelona Esquerra (CAPSBE) Barcelona Spain; ^4^ Primary Healthcare Transversal Research Group Institut d'Investigacions Biomèdiques August Pi i Sunyer (IDIBAPS) Barcelona Spain; ^5^ Doctoral Programme in Medicine and Translational Research University of Barcelona (UB) Barcelona Spain; ^6^ Departament de Salut Pública, Salut Mental i Maternoinfantil, Facultat d'Infermeria Universitat de Barcelona Barcelona Spain; ^7^ Gerència d'Atenció Primària i a la Comunitat Baix Llobregat Institut Català de la Salut Cornellà Spain; ^8^ Fundació Institut Universitari per a la Recerca a l'Atenció Primària de Salut Jordi Gol i Gurina (IDIAPJGol) Barcelona Spain

**Keywords:** cardiac rehabilitation, gender identity, myocardial infarction, primary health care, qualitative research

## Abstract

**Aim:**

To explore, including from a gender perspective, how people who have suffered a myocardial infarction describe the changes they need to adopt during the recovery phase following hospital discharge.

**Design:**

Qualitative interpretive phenomenological study.

**Method:**

Purposive theoretical sampling strategy with maximum variation. Semi‐structured interviews were conducted with subsequent thematic analysis of semantic content, with deductive coding based on relevant aspects identified in the scientific literature and inductive coding on emerging codes in interviewees' discourse.

**Data Sources:**

The interviews were conducted between March 2022 and December 2023.

**Results:**

The participants had an average age of 61 years. A total of nine participants were interviewed. Four of these were women. No one identified as non‐binary. Three themes were identified: initiation of one's own resources, engagement with one's own emotions, and follow‐up by the health team.

**Conclusion:**

In this sample, both men and women initially described motivation to assume responsibility for their self‐care and lifestyle changes. Women reported greater difficulties in returning to work and maintaining physical activity, which may contribute to increased vulnerability during recovery. These findings may help inform nurse‐led cardiac rehabilitation programmes that respond to patients' real needs.

## 1 Introduction

The association between acute coronary syndrome risk factors and gender suggests that people with roles more closely associated with femininity may face a higher burden of risk factors, regardless of sex. However, the cardiac rehabilitation phase has rarely been explored from a gender perspective.

## 2 Background

Cardiovascular disease is the most common cause of death in Europe and accounts for 38% of all deaths in women and 44% in men (Timmis et al. 2022). However, incidence among women is underestimated, with greater increases and worse outcomes (Ministry of Health 2022; Smolderen et al. 2019).

During the post‐infarction recovery period, the aim is to minimise physiological and psychosocial effects, control symptoms and reduce the risk of future cardiovascular events. With these goals in mind, recommendations regarding behavioural changes in eating, arterial pressure control, relaxation, weight control, cessation of alcohol and tobacco use, and adaptation to international guidelines on progressive exercises are made (Visseren et al. 2021), although they are not gender‐specific (Gulamhusein and Ahmed 2024).

Sex biases in clinical presentation, diagnosis and referral for intervention following ischemic heart disease have been widely explored (Solola Nussbaum et al. 2022). Nevertheless, while sex differences are usually associated with biological aspects, the gender construct has typically been used to understand accepted and attributed social roles along the masculinity/femininity continuum (Mauvais‐Jarvis et al. 2020). The link between acute coronary syndrome risk factors and gender indicates that genders more closely associated with femininity bear a heavier burden of risk factors, particularly in the young population, regardless of sex. However, the literature reveals that women disproportionately cope with multiple demanding roles, gender pay gaps and a higher number of single‐parent families. Being the main breadwinner and assuming responsibility for housework have a considerable impact on risk factors (Ebong et al. 2024). However, while existing evidence has described sex‐ and gender‐related inequalities in cardiovascular risk, diagnosis and treatment, less is known about how people themselves experience the behavioural and psychosocial changes required during recovery after myocardial infarction. This gap is particularly relevant because current recommendations for post‐infarction recovery are not gender‐specific and may not fully account for the influence of gendered social roles, care responsibilities, employment conditions and socioeconomic vulnerability on the ability to adopt and maintain lifestyle changes. Exploring these experiences from a gender perspective may therefore help to identify recovery needs that remain insufficiently addressed in routine follow‐up and cardiac rehabilitation.

From a nursing perspective, understanding these experiences is particularly relevant because nurses are often involved in post‐discharge education, symptom monitoring, lifestyle counselling, and continuity of care. Identifying how gendered social roles, employment conditions, comorbidities, and family responsibilities affect recovery may enable nurses to provide more individualised, equitable, and context‐sensitive support after myocardial infarction (Lizcano‐Álvarez et al. 2023).

## 3 The Study

### 3.1 Aim and Objective

To explore, including from the gender perspective, how people who have suffered a myocardial infarction describe the changes they need to adopt during the recovery phase following hospital discharge, in order to identify needs that may inform person‐centred nursing assessment, follow‐up and support.

## 4 Methods

### 4.1 Design and Theoretical Framework

This was a qualitative interpretative phenomenological study. This approach highlights differences in how people experience and understand their health condition and needs, allowing these experiences to inform clinical interventions (Smith et al. 2022).

The study was informed by interpretative phenomenology, which assumes that reality is constructed through individuals' perceptions and interpretations of their experiences. This framework is particularly suited to exploring subjective aspects of health and illness and how people construct their recovery narratives in response to medical, emotional, and social expectations.

### 4.2 Setting, Sampling and Recruitment

A purposive theoretical sampling strategy with maximum variation was used to diversify discursive positions (Villamin et al. 2024). Sample profiles were defined by gender (man, woman, non‐binary), age (18–40, 40–60 and > 60 years), employment status (active or not active) and socioeconomic situation, according to the interprofessional minimum wage in Spain.

Participants were recruited from two Primary Healthcare Centres (PHC) located in an urban area in Spain, where they were undergoing post‐infarction follow‐up. Eligibility was determined according to predefined inclusion and exclusion criteria, which are presented in Table 1.

**TABLE 1:** Inclusion and exclusion criteria for participant recruitment.

Inclusion criteria	Exclusion criteria
Adults aged ≥ 18 years Hospital admission for acute coronary syndrome, according to ICD‐10 diagnostic codes I20–I22 Discharge home within the catchment area covered by the selected Primary Healthcare Centres in an urban area of Spain	Acute aortic disease, severe pulmonary arterial hypertension, uncontrolled arrhythmias, decompensated heart failure, or severe valvular or congenital heart disease. Previous cardiac valve and/or septal surgery Non‐cardiac diseases or conditions preventing exercise practice Severe mental disorder, psychiatric condition, cognitive impairment or dementia preventing participation in an in‐depth interview. Verbal communication problems, including aphasia or insufficient language proficiency to communicate effectively Previous participation in a hospital‐based cardiac rehabilitation programme

The recruitment process followed a chronological sequence. First, potentially eligible patients were identified by the healthcare professionals involved in post‐infarction follow‐up at the participating PHC centres, according to the inclusion and exclusion criteria. Patients who met the eligibility criteria were then approached during their follow‐up care and were briefly informed about the study. Those who expressed initial interest were contacted by a member of the research team, who provided detailed oral and written information about the study aims, the voluntary nature of participation, confidentiality, and the option of withdrawing at any time without any effect on their healthcare.

Recruitment continued until data saturation was reached, understood as the point at which no new themes or insights emerged from the interviews. Although the final sample comprised nine participants, this number was considered sufficient given the specificity of the study objectives and the relative homogeneity of the participant group. This is consistent with prior research indicating that thematic saturation in qualitative studies is often achieved with small, focused samples (Hennink and Kaiser 2022).

### 4.3 Data Collection

Two authors, both experienced in qualitative research and with no prior relationship with the participants or involvement in their direct healthcare, conducted the interviews between March 2022 and December 2023. Interviews were conducted in the language of the participants' choice, either at the participants' home or in a designated private room and lasted approximately one hour.

Consistent with the qualitative interpretative phenomenological design, the study did not define clinical endpoints. Instead, the phenomenon of interest was participants' lived experience of cardiac recovery after hospital discharge, with particular attention paid to perceived lifestyle changes, emotional responses, social and family roles, work readaptation, sexuality, and follow‐up by the healthcare team.

All interviews were audio‐recorded and transcribed verbatim, capturing both verbal content and relevant non‐verbal cues.

### 4.4 Data Analysis

The interview transcripts constituted the textual corpus for the analysis. A thematic analysis informed by an interpretative phenomenological approach was conducted, combining deductive and inductive coding. Deductive codes were derived from relevant themes identified in the scientific literature, while inductive codes emerged directly from participants' narratives.

The analytical procedure included the following stages: (1) repeated reading of the transcripts to generate initial analytical impressions and identify meaningful text segments; (2) software‐aided coding using ATLAS.ti; (3) review of the internal consistency of each code, category, and subcategory; (4) review of consistency across categories; and (5) development of the results through code and category maps, explanatory texts, and the selection of verbatim quotations to illustrate the findings.

The initial analysis was conducted by two authors with expertise in qualitative methodology. Themes and subthemes were subsequently refined through constant comparison and discussion among the research team until consensus was reached. Preliminary findings were discussed with all researchers, including three experts in Primary Healthcare follow‐up and one researcher with expertise in gender perspective. Disagreements were resolved through a consensus‐negotiation process.

### 4.5 Ethical Considerations and Rigour

This study was conducted in accordance with the principles of the Declaration of Helsinki and was approved by the relevant Research Ethics Committees. All participants provided written informed consent before the interview. Participants were assigned alphanumeric codes, and direct personal identifiers were removed from the transcripts. As age, gender, and other contextual characteristics were retained for analytical purposes, the data were pseudonymised rather than fully anonymised. Confidentiality was protected throughout the research process.

To ensure the trustworthiness of the findings, the criteria of credibility, transferability, confirmability, and dependability were applied (Lincoln and Guba 1985). The SRQR (Standards for Reporting Qualitative Research) checklist (O'Brien et al. 2014) guided the reporting of the study and is provided as File S1. Credibility was strengthened through participant validation of interview transcripts and continuous reflexive dialogue among researchers to minimise bias and support coding consistency. Transferability was supported by purposive sampling with maximum variation and detailed contextual descriptions. Confirmability was enhanced through the use of verbatim quotations and external peer review of the coding framework by a researcher with expertise in gender perspective. Dependability was supported by documenting analytical decisions and involving researchers who had not participated in data collection.

## 5 Findings

### 5.1 Participants

A total of nine participants were interviewed. Four participants were women and five were men. No participant identified as non‐binary. Ages ranged from 44 to 78 years, with a similar average age between genders. Although both centres were in a neighbourhood generally characterised by a high socioeconomic profile, the final sample also included participants with limited economic resources, thereby introducing some degree of socioeconomic diversity. Participants' characteristics are presented in Table 2.

**TABLE 2:** Sample characteristics.

	Gender	Age	Personal income according to IMW	Occupation	Living with	Marital status	Date of myocardial infarction	Date of interview
P1	Man	71	+1.5	Retired, pharma' company worker	Partner	Married	1 Jan 2019	22 Jun 2022
P2	Woman	78	1.5	Retired nurse	Alone	Widow	1 Jan 2019	1 Jul 2022
P3	Man	60	1.5	Active Waiter	Partner and adult daughter	Married	24 Oct 2017	4 Jul 2022
P4	Woman	58	1.5	Waitress^a^	Partner	Married	23 Jul 2019	21 Jul 2022
P5	Man	69	+1.5	Retired Geriatrician	Partner	Married	1 May 2017	17 Oct 2022
P6	Woman	44	0.5	Nurse^a^	Partner	Married	1 Jan 2015	17 Oct 2022
P7	Man	51	+1.5	Sick leave Auditor	Partner and two school‐age children	Married	2 Jul 2023	28 Nov 2023
P8	Woman	61	No income	Unemployed Care worker	Daughter	Widow	11 Oct 2023	19 Dec 2023
P9	Man	57	1.5	Active Family company	Partner and 2 adult offspring	Married	30 Jul 2023	19 Dec 2023

*Note:* IMW, Interprofessional Minimum Wage in Spain is €15,876 per annum.

^a^ Currently in a recognised work disability status.

### 5.2 Thematic Analysis and Main Themes

Thematic analysis identified three core themes that illustrate participants' experiences and care needs during the recovery process after myocardial infarction. These themes reflect not only the challenges of physical and emotional recovery but also the ways in which gender roles and expectations shaped the participants' experiences and coping strategies (see summary in Table 3).

**TABLE 3:** Table of themes and subthemes.

Theme	Subtheme	Definition of subtheme
1. Initialisation of one's own resources: This theme gathers participants' reflections on their lifestyle prior to the heart attack. This entails asking about how their previous habits may have played a role in the heart attack. It is closely linked to participants' need to establish new priorities, set limits, reduce stress and adopt more healthy habits, such as dietary improvements, smoking cessation and adjusting the burden of work. Requires investment of time and effort to modify lifestyle, prioritising oneself, recognising one's own needs and being free of certain obligations and responsibilities	1.1 Responsibility for self‐care	Assuming lifestyle responsibilities
	1.2 Limitation due to comorbidities and secondary effects of the heart attack	Aspects of the disease which limit daily life activities
	1.3 Readaptation to working life	Adaptation to new limitations that can produce physical, psychological and economic challenges
	1.4 Household management	References to home management and organisation after the heart attack
	1.5 Sexual relations	Adaptations after the heart attack
	1.6 Family support and social life	Support from family members, friends and colleagues
2. Engagement with one's own emotions: this theme gathers information on which emotions emerge after the heart attack and how participants express, manage, modulate and control them	2.1 Guilt	Guilt with regard to one's lifestyle prior to the heart attack
	2.2 Fear	Experience of fear, uncertainty, fear of death and loneliness
3. Follow‐up by the Health Care Team: Includes how the participants express themselves regarding the actions carried out by the health team after the heart attack, and their unmet needs. This category focuses on testimonies about follow‐up during the first year of recovery after the heart attack	3.1 Initial recommendations	Narratives about health service recommendations
	3.2 Follow‐up	Narratives related to Acute Myocardial Infarction follow‐up after hospital discharge

Within each theme, shared experiences between men and women are distinguished from gender‐related patterns observed in the sample. These gender‐related patterns mainly concerned physical activity, return to work, household responsibilities, and the continuity of care perceived during recovery.

#### 5.2.1 Theme 1: Initiation of One's Own Resources

This theme explores how participants mobilised personal and behavioural resources to manage lifestyle changes following the cardiac event. Gender roles influenced the perceived capacity to implement and maintain such changes in relation to self‐care, work, household responsibilities, sexuality, and social habits.

##### 5.2.1.1 Responsibility for Self‐Care

Participants described the early phase of recovery as a period of heightened motivation to adopt healthier behaviours, particularly regarding diet and alcohol consumption. However, participants described that adherence to these changes varied during the period between hospital discharge and the interview. For instance, one male participant noted that while he had reduced alcohol intake, he still maintained some pre‐existing habits:

Before, I drank more, now I have a little glass of beer. Then, when I finish work, a couple of glasses of beer… on Sunday when we leave here, maybe a barbeque or a bottle of wine or cava and then… well, a gin and tonic, but no more. (P3, man, 60 years)

In terms of physical activity, walking was the most frequently mentioned activity and was perceived as accessible and manageable for most participants. However, some described physical limitations that hindered their ability to maintain regular routines. A female participant reflected on her difficulties adhering to exercise recommendations:

Now, I just, as they say, walk… I'm not going to lie to you and say that I do it as a daily exercise. I walk because I go out and I have to walk… my limitations are because of the knee problem. (P8, woman, 61 years)

For others, the availability of time during recovery facilitated the adoption of new routines. One man expressed how having more time allowed him to integrate walking into his daily routine: ‘Because I have more time, and because they said I had to walk a lot and I walk’ (P7, man, 51 years). Overall, while men more often described walking as an activity that could be incorporated into their daily routine, women more frequently linked difficulties in maintaining physical activity to fatigue, comorbidities and limitations in mobility.

##### 5.2.1.2 Limitations Related to Comorbidities and Post‐Infarction Symptoms

For many women, the impact of comorbidities outweighed the consequences of the heart attack itself. These conditions, often pre‐existing, continued to restrict physical activity and dietary habits, resurfacing throughout the interviews each time participants discussed lifestyle changes. One woman described how her capacity for movement remained limited due to ongoing joint problems:

I had a total hip replacement, and now in January or February they'll do the other one, because both are very damaged. I have arthrosis… my limitations are because of the knee problem. (P8, woman, 61 years)

Fatigue and persistent tiredness since the heart attack were also recurrent themes, particularly among women in this sample. Participants reported feeling less energetic, often needing to rest after minimal exertion. A 44‐year‐old woman contrasted her current state with her previous routine, explaining how she now becomes breathless after simple tasks: ‘Now, of course, I get much more tired than before. Before I could go here and there all day, now I can't. Now you go, ah, wait… yes, yes, you get breathless’ (P6, woman, 44 years).

Similarly, an older participant noted how her ability to climb stairs had significantly diminished: ‘Only that since then, I get tired more. For example, going up stairs. I think about it a bit because I puff a lot’ (P2, woman, 78 years).

##### 5.2.1.3 Readaptation to Working Life

In this sample, men generally reported returning to work without requiring significant adjustments. In contrast, women described substantial difficulties in re‐entering the workforce, often as a direct consequence of the heart attack and in the context of more precarious employment situations. For some, the event led to job loss, compounding pre‐existing vulnerabilities associated with precarious employment. One woman expressed the impact of being dismissed after the event: ‘They sacked me and now I cope as best I can’ (P4, woman, 58 years).

For others, the perception of being a less reliable employee due to ongoing health issues further complicated their job search. A younger participant described how potential employers viewed her health status as a barrier to employment:

Nobody wants to give you a job as they don't see you as being 100%… and they say this one will be on sick leave more… or more time at home than working. Now you don't even look. For what? (P6, woman, 44 years)

##### 5.2.1.4 Household Management

For women, the distribution of household chores temporarily shifted after the heart attack, with family members or partners initially taking over some tasks. However, this support was often short‐lived, as both the women and their families perceived that they could soon resume their usual responsibilities. One woman described how, despite receiving some help initially, the household routine quickly returned to its previous state:

At first my husband and children didn't let me do anything, sit quietly, relaxed. I was doing the food little by little and, well, little by little each day you do a little bit more. But the routine. In no time it's back to normal. (P4, woman, 58 years)

In contrast, men reported minimal changes in their household roles, stating that their spouses continued to take on most domestic tasks, with no significant adjustments during the recovery period. This contrast suggests that recovery occurred within pre‐existing gendered patterns of household responsibility.

##### 5.2.1.5 Sexual Relations

Sexually active participants reported no major changes in their sexual activity following the heart attack, although some men noted a decrease in frequency. This reduction was often attributed to age or the potential side effects of medication, but few sought medical advice on the matter. One man reflected on how his libido had declined, associating it with both medication and advancing age:

I don't know if it's because of the pills or what, but the sex‐appeal, that's gone. I don't know… Well, it could be all the medication… we're getting on now, we're already 60 years old. If this had happened to me when I was 40, well, then I'd be worried… We're doing fine. (P3, man, 60 years)

##### 5.2.1.6 Family Support and Social Life

Family members and friends initially adjusted their behaviour to support participants in maintaining lifestyle changes, particularly regarding smoking cessation. One man noted how those around him made efforts to shield him from temptation in the early stages of recovery: ‘Everyone around me smokes… At first, they would nip around the corner so I wouldn't see it’ (P3, man, 60 years).

These findings, identified within the theme Initiation of One's Own Resources, highlight the nursing role in assessing patients' capacity and barriers to self‐care, providing individualised lifestyle education, and supporting realistic changes adapted to functional limitations, family responsibilities, and employment circumstances.

#### 5.2.2 Theme 2: Engagement With One's Own Emotions

This theme explores the emotional impact of experiencing a myocardial infarction, particularly how participants processed fear, guilt, and vulnerability during recovery. Emotional responses were shaped not only by the cardiac event itself but also by personal history, family roles, and perceptions of risk.

##### 5.2.2.1 Guilt

Receiving information about heart‐healthy lifestyles led participants to reflect on their previous behaviours, often resulting in feelings of guilt. Some attributed their condition to personal lifestyle choices made prior to the heart attack. One woman, aged 44, expressed regret over not having been more attentive to her health before the event:

I thought about how bad I'd been before. If I had behaved better, if I'd taken a bit more notice… then maybe I wouldn't be like this, but I asked for it. (P6, woman, 44 years)

##### 5.2.2.2 Fear

Participants expressed a persistent sense of vulnerability related to the possibility of a new cardiac event. For some, this fear was heightened by witnessing similar incidents in others. One man, for instance, described how the sudden death of a friend during a sports activity deeply affected him:

I'm left with the question of why, and whether it's going to happen to me again… two years ago a friend of mine… we were playing padel and he died on the court. (P7, man, 51 years)

Fear also extended into daily life, such as sleeping or leaving home without medication. A woman recalled the anxiety she experienced in the weeks after the event: ‘At first I was really scared to fall asleep, that it could happen to me again and I wouldn't wake up’ (P4, woman, 58 years).

Family members also shared this fear, sometimes monitoring participants closely to ensure their safety.

Within the theme *Engagement with One's Own Emotions*, these accounts indicate that nurses can assess fear, guilt, and perceived vulnerability during follow‐up, provide initial emotional support, and identify patients who may require referral for specialised psychological care.

#### 5.2.3 Theme 3: Follow‐Up by the Healthcare Team

This theme focuses on participants' perceptions of the healthcare team's involvement after hospital discharge. While initial emergency care was generally described as prompt and effective, post‐discharge follow‐up was perceived as insufficient, inconsistent, or unclear, particularly regarding lifestyle recommendations and professional roles. Participants referred mainly to medical follow‐up, while nursing follow‐up was mentioned less frequently in their accounts.

##### 5.2.3.1 Initial Recommendations

Participants generally agreed that emergency care was prompt and effective, but they described the discharge recommendations as sparse and vague, especially regarding lifestyle changes. Advice on diet, smoking, or exercise was considered basic or insufficient. One man highlighted the simplicity of the message:

The only thing they said to me was… watch the diet, grilled and eat like that, things with no fat and stuff like that. But nothing else. I carry on living my normal life as usual… The only thing was I gave up smoking, and that's it. (P3, man, 60 years)

Others mentioned they received no advice at all before discharge: ‘On the hospital ward; any recommendations before leaving?—No, none—’ (P2, woman, 78 years).

Unclear instructions about driving or returning to work generated confusion. A participant questioned the rationale behind not being allowed to drive: ‘They told me I couldn't drive… but if I'm a front‐seat passenger? Isn't there an airbag too?’ (P9, man, 57 years).

##### 5.2.3.2 Follow‐Up

Participants described post‐discharge follow‐up as inconsistent and poorly coordinated. Many expected early contact with a cardiologist but instead experienced long delays or no structured plan. One man expressed frustration: ‘I don't even know who the cardiologist is’ (P3, man, 60 years).

Several participants noted that follow‐up in primary care focused on other chronic conditions, not post‐infarction recovery. A woman explained: ‘Well, the nurse followed me up for diabetes’ (P2, woman, 78 years). Some participants sought reassurance from private specialists due to the lack of system feedback. One man explained: ‘Maybe if there was a call… Hey, how are you feeling? It's more psychological… I went to a private cardiologist… he told me not to worry’ (P7, man, 51 years).

Others compared the cardiac follow‐up unfavourably with previous experiences in other health services: ‘I had a cancer in 2021 and I had a direct telephone number I could call… There was no telephone here’ (P9, man, 57 years). Participants' experiences within the theme Follow‐Up by the Healthcare Team highlight the nurse's role in reinforcing discharge education, assessing informational and support needs, and facilitating continuity between hospital care, primary care, and cardiac rehabilitation.

## 6 Discussion

The study aimed to explore people's experiences during recovery after myocardial infarction in primary care, while examining how gender roles and expectations shaped the perceived changes required after hospital discharge. Our findings suggest that participants who had experienced a myocardial infarction, both men and women, were initially motivated to assume responsibility for their self‐care. Faced with the perception of the risk of a new cardiac event, both men and women modify risky lifestyles and avoid stress in their usual routines. They follow initial health advice regarding food, alcohol, tobacco, and exercise; the pillars of international recommendations (Visseren et al. 2021). However, they show a tendency to return to routines that incur risk. Among the habits that are maintained, only self‐reported exercise (typically walking) and abstinence from smoking persist over time. There is evidence that giving up smoking is the factor that most contributes to a decrease in mortality from cardiovascular disease (Buigues et al. 2022). These findings contrast with the results of the study by Kotseva et al. (2019), where the smoking habit is one of the most challenging to modify. In this sample, women described greater difficulty in maintaining physical activity, mainly in relation to post‐infarction symptoms or other comorbidities. Comorbidities were mentioned in other studies as negatively impacting participants' ability to perform and enjoy physical activity (Coull and Pugh 2021). Men in this sample described setting aside time for walking consistent with previous evidence suggesting that men may exercise more regularly after myocardial infarction (Kotseva et al. 2019). These findings suggest that behavioural change after myocardial infarction was not experienced only as an individual health‐related task, but also as a process shaped by gendered social roles, employment conditions, domestic responsibilities and the availability of time and support for self‐care. Various authors discuss responsibility for the initial changes and the difficulty of maintaining good habits over the long term (Coull and Pugh 2021). In Europe, the EUROASPIRE study revealed that most coronary patients, men and women, normally exhibit low adherence to heart‐healthy recommendations (Kotseva et al. 2019). People have the potential to change but to convince them to do so, it is essential to apply behavioural theories and to take social, cognitive, affective and environmental factors into consideration (Kotseva et al. 2019). This required support period has been shown to be longer for women (Firoozabadi et al. 2023).

With respect to the return to work, the men interviewed described resuming their previous occupations, whereas women in this sample reported greater difficulties, often in the context of more precarious economic and employment circumstances, the consequences of which are related to worse outcomes during the first year following the heart attack, including effects on physical and mental health, quality of life and the possibility of relapse. Perhaps owing to their comorbidities, perceived as aspects which shape their lives, women show greater fatigue, even revealing insecurity over their ability to cope with their future role on low income. Being a woman is a predictor of inability to work after acute myocardial infarction (Barresi et al. 2024).

Regarding household management, participants' accounts suggested that pre‐existing domestic roles tended to persist during recovery. From a gender‐role perspective, these findings suggest that cardiac recovery was embedded within pre‐existing social expectations regarding masculinity and femininity. In this sample, men's narratives tended to emphasise continuity in paid work and fewer changes in household responsibilities, whereas women described recovery within a context of accumulated demands, including fatigue, comorbidities, employment insecurity and the rapid resumption of domestic tasks. These patterns may reflect how traditionally feminine roles, particularly responsibility for care and household management, can reduce the time and energy available for sustained self‐care after myocardial infarction. Conversely, traditionally masculine expectations related to work continuity and autonomy may contribute to a perception of faster return to normality, while potentially limiting the expression of vulnerability or emotional needs. Although these interpretations cannot be generalised, they help explain how gendered social roles may influence the capacity to adopt and maintain behavioural changes during cardiac recovery. The usual roles within the femininity‐masculinity binomial, although they are slightly modified in the period immediately following the heart attack, are quickly reestablished. There was no evidence that the men in our study adopted roles traditionally associated with the feminine gender. Examination of these aspects is fundamental in prevention, as the characteristics typically attributed to women increase, especially among the young. The risk of adverse cardiovascular outcomes regardless of sex is linked to stress and overload arising from caring for the family and the home (Ebong et al. 2024).

Discourse revealed that social representations associated with sexuality are complex and that the age factor is important where men are concerned. Although most people usually expect advice on sexual activity, this did not happen in the majority of situations in our study. Failure to talk about the subject on the part of professionals can have important repercussions on the relationship and self‐esteem as there may already be a reluctance to seek medical advice on this issue for cultural reasons (Arıkan and Yavaş 2024).

People recognise that the support of family and friends is important, particularly in the early stage of recovery. However, there are no comments on the role of the family as a resource for changing habits, which are observed in other studies (Smolderen et al. 2019). Culturally, gatherings in Spain are held in settings around food, alcohol and tobacco. Maintaining healthy eating or abstaining from alcohol or tobacco means that these social events emerge as decisive moments in which they must put strong convictions into practice to avoid certain consumption and not everyone is able to achieve this. Changing lifestyle habits is a multifaceted process influenced by many factors, with sociocultural matters of fundamental importance (Tunsi et al. 2023).

Participants draw on previous experiences to assess what to do or avoid, recognising their responsibility for aspects such as tobacco, poor diet or stress, believing that they did not take good enough care of themselves. For both genders, the heart attack represents a turning point, a ‘wake‐up call’ that reminds them of their vulnerability. People experience conflicting emotions: fear due to the disruption to their lives after the heart attack and gratitude at having survived. However, it is also noted that routine and forgetting about the process affects adherence to long‐term recommendations with the passage of time. Uncertainty is another emotion that appears directly after discharge and is related to unmet needs arising from unclear, superficial instructions, or a complete absence of them, on questions such as when to resume driving or when to return to work. Recent research has shown that this uncertainty contributes to ongoing emotional distress for several months post‐discharge, especially when communication with healthcare professionals is insufficient (Dreyer et al. 2021).

Patients' personal perspectives on what they need in the process seem to be overlooked, with a focus on medical care and clinical recovery in the early stages. This can have an impact on well‐being and initial levels of anxiety which may, in turn, affect the recovery process. A recent qualitative study of cardiac rehabilitation practitioners found that, although they recognised patients' complex psychological needs—including anxiety, adjustment difficulties, and cardiac‐related worries—their degree of confidence in providing mental health support varied, and many services still lack comprehensive psychological provisions (Wray et al. 2024).

Nevertheless, patients tend to excuse the healthcare system for not providing this type of guidance, acknowledging that it is overburdened and focused primarily on immediate survival rather than on long‐term preventive care. A recent European study found that patients and their relatives often perceived healthcare professionals as lacking time to talk with them or address their emotional and informational needs following myocardial infarction (Qin et al. 2022).

In this study, unmet emotional and informational needs were described by both men and women, although the psychological changes brought about by the new situation were reflected in the greater importance given to meaningful personal relationships. Patients should be automatically and systematically referred to cardiac rehabilitation at the time of discharge and thus could receive all the information necessary to begin the programme. The main issue is the limited availability of such programmes within our healthcare system (Qiu 2024).

The patients in our study received care to monitor biological risk markers and other tests (echocardiogram, electrocardiograms, etc.) but are not satisfied with the information provided or the continuity of care, as previously indicated in the study by Bårdsgjerde et al. (2020). Most have not started a cardiac rehabilitation programme since their myocardial infarction, which may increase the risk of poorer secondary prevention outcomes (O'Toole et al. 2020). Sequential, multidimensional interventions should focus on individual needs, following a holistic assessment of each case, including the family in follow‐up process management (Brown et al. 2024; Goldfarb et al. 2022).

Participants' experiences, reflected in the three themes identified through the analysis, highlight opportunities for nurse‐led intervention. Nurses could assess readiness for change and barriers to self‐care, use motivational interviewing to establish realistic goals, and provide tailored education on medication, symptom recognition and lifestyle changes. Follow‐up should also assess emotional well‐being, provide initial psychosocial support and facilitate referral when specialised care is required. With the patient's agreement, relatives could be involved in education and goal setting to address family concerns and support sustainable change. Early nursing contact and coordination between hospital care, primary care and cardiac rehabilitation may strengthen continuity of care. These interventions should be adapted to functional capacity, comorbidities, family responsibilities, employment circumstances and available resources (Buigues et al. 2022; Dreyer et al. 2021; Lizcano Álvarez et al. 2021; Qin et al. 2022; Qiu 2024; Wray et al. 2024).

Mobile health (mHealth) technologies may represent a complementary strategy to support continuity of care and long‐term adherence after myocardial infarction, particularly when access to structured cardiac rehabilitation programmes is limited. Although our findings do not directly evaluate digital interventions, participants' accounts of insufficient follow‐up, uncertainty after discharge and the difficulty of maintaining lifestyle changes suggest potential areas where smart devices and mobile applications could provide continuous monitoring, motivational reinforcement and personalised feedback. Evidence from the LIGHT randomised clinical trial and its sub‐studies has shown that mobile applications and smart devices may contribute to improvements in physical activity, heart rate variability and cardiorespiratory fitness in patients at high cardiovascular risk (Hayiroǧlu, Çinar, et al. 2021; Hayiroǧlu, Çinier, et al. 2021; Hayıroğlu et al. 2023).

In the Spanish context, where healthcare is publicly funded and largely provided through the public health system, the implementation of digital health tools also needs to be considered in terms of economic sustainability, equity of access and feasibility within routine care. Budgetary constraints may limit the widespread adoption of certain technologies, particularly when they require specific devices, platforms or additional professional time. At the same time, gaps in digital literacy, knowledge of eHealth services and the usability of mobile platforms may restrict their uptake among more vulnerable populations. Smartphones may therefore play a key role in democratising access to digital health services, provided that these tools are affordable, accessible and adapted to users' needs (Mahou et al. 2021). Otherwise, digital innovation may reinforce, rather than reduce, existing socioeconomic inequalities. For this reason, mHealth tools should be considered as an adjunct to, rather than a replacement for, nurse‐led and gender‐sensitive cardiac rehabilitation programmes.

### 6.1 Strengths and Limitations of the Work

Some limitations should be considered when interpreting the findings. First, although the sample size was consistent with the interpretative phenomenological approach and data saturation was reached, the inclusion of nine participants may limit the transferability of the findings to other contexts. In addition, participants were recruited from two Primary Healthcare Centres located in neighbourhoods with high socioeconomic indicators in the study area; therefore, experiences of people from more socioeconomically disadvantaged areas may be under‐represented.

Second, although the sampling strategy included the possibility of recruiting people with non‐binary gender identities, all participants identified as either men or women. Consequently, the gender analysis was limited to the experiences, roles and expectations described by men and women in this sample. Nonetheless, analyses centred on gender roles and expectations, allowing us to understand how social constructions influence the experience of illness and recovery.

Finally, given that some interviews were conducted retrospectively, recall bias cannot be completely ruled out. However, the use of in‐depth interviews, verbatim transcription, and participant validation contributed to strengthening the credibility of the findings.

Another important factor was the first interviewees' lack of participation in cardiac rehabilitation programmes. Initially, the research team had linked this to the impact of the COVID‐19 pandemic, which transformed health service provision and promoted telecare. As a response, inclusion criteria were modified to incorporate people who had a heart attack once normal health services had resumed. However, neither the new interviewees had participated in such programmes, suggesting their absence in the study area.

### 6.2 Recommendations for Further Research

Further research should focus on evaluating long‐term adherence to heart‐healthy behaviours after myocardial infarction, particularly examining the influence of gender, social roles, and emotional responses. It is essential to explore how behavioural theories can be effectively applied in designing tailored interventions that address not only physical but also psychosocial and environmental factors. Special attention should be paid to the experiences of women, who face more barriers to physical activity and returning to work, and to integrating family support as an active element in secondary prevention. Research should also examine strategies for improving communication on sensitive topics, such as sexuality, and better addressing patients' psychological needs during recovery. Furthermore, the effectiveness of nurse‐led, motivational and interdisciplinary approaches at the primary care level should be assessed in improving continuity of care, patient satisfaction, and uptake of cardiac rehabilitation programmes, especially for populations at higher risk of relapse due to social or gender‐related vulnerabilities.

### 6.3 Implications for Nursing Practice

Nursing assessment after myocardial infarction should be gender‐sensitive and individualised rather than based on assumptions associated with sex or gender categories. Nurses should assess functional capacity, fatigue, comorbidities, emotional well‐being, household and caregiving responsibilities, employment circumstances, family support and the practical feasibility of recommended lifestyle changes. Based on this assessment, an individualised care plan should establish realistic goals for physical activity and self‐management, identify support required for returning to work and daily responsibilities, and include scheduled follow‐up and referral when additional psychological, social or rehabilitation support is needed. Particular attention should be paid to vulnerabilities more frequently described by women in this sample, including fatigue, mobility limitations, employment insecurity and the rapid resumption of household responsibilities, while recognising that these needs may also affect men and should be assessed individually.

## 7 Conclusion

Following analysis of roles and gender expectations in the process of recovery after myocardial infarction, our findings suggest that both men and women were initially motivated to take responsibility for their self‐care. In this sample, women described greater economic vulnerability related to difficulties in returning to work, as well as lower adherence to physical activity recommendations. These aspects appeared to be linked mainly to pre‐infarction morbidity, even though women did not constitute an older group than men. These findings indicate that women may experience specific vulnerabilities during cardiac recovery that should be considered in nurse‐led, gender‐sensitive rehabilitation programmes. Nurses are well placed to translate these findings into individualised care by strengthening holistic assessment, discharge planning and continuity of follow‐up. Nurse‐led care should combine assessment of functional, emotional and social needs with tailored patient education, self‐management support, brief emotional support and coordination between hospital care, primary care and cardiac rehabilitation.

A gender‐sensitive nursing approach should not rely on assumptions based solely on sex or gender categories, but should explore how social roles, comorbidities, and socioeconomic circumstances shape each person's opportunities for self‐care. Integrating these dimensions into individualised care plans, post‐discharge follow‐up and cardiac rehabilitation may contribute to more continuous, person‐centred and equitable recovery after myocardial infarction.

## Author Contributions

**Susana Santos‐Ruiz:** conceptualization, funding acquisition, formal analysis, data curation, writing – original draft, writing – review and editing, investigation, methodology. **Carmen Jerez‐Molina:** conceptualization, methodology, investigation, data curation, formal analysis, writing – original draft, writing – review and editing. **Rocio Rodriguez‐Romero:** investigation, resources, project administration, writing – review and editing. **Juan José Zamora‐Sánchez:** writing – review and editing. **Luis González‐De Paz:** conceptualization, methodology, supervision, writing – review and editing.

## Funding

This study was supported by the Official College of Nurses of Barcelona (https://www.coib.cat/ca‐es/inici.html) as part of the Nurse Research Projects Grants (PR‐503/2021).

## Ethics Statement

This study was conducted in accordance with the Declaration of Helsinki and was approved by the relevant Research Ethics Committees: the Comité de Ética de la Investigación con medicamentos (CEIm) de la Fundació Sant Joan de Déu (PIC‐137‐21) and the Comité de Ética de la Investigación con medicamentos (CEIm) del Hospital Clínic de Barcelona (HCB/2022/0276).

## Consent

All participants provided written informed consent before participation.

## Conflicts of Interest

The authors declare no conflicts of interest.

## Supporting information


**File S1:** Completed Standards for Reporting Qualitative Research (SRQR) checklist for this study.

## Data Availability

The qualitative data that support the findings of this study are not publicly available because they contain sensitive information that could compromise participant confidentiality. Access to appropriately de‐identified data may be considered upon reasonable request to the corresponding author, subject to ethical and institutional approval.
