# Supplementary material for: Users' Experiences During Cardiac Recovery Following Acute Myocardial Infarction: A Qualitative Study With a Gender Perspective
Source: Nurs Open. 2026 Oct 16;13(10):e70886. doi: 10.1002/nop2.70886 (PMC13675514; doi:10.1002/nop2.70886)
Supplement: Supplementary file 1 [file NOP2-13-e70886-s001.docx]

|  | **Standards for Reporting Qualitative Research (SRQR)*** |  |
| --- | --- | --- |
|  | <http://www.equator-network.org/reporting-guidelines/srqr/> |  |
|  |  | **Page/line no(s).** |
| **Title and abstract** | |  |
|  | **Title** - The title “Users’ experiences during cardiac recovery following acute myocardial infarction. Qualitative study with a gender perspective.” identifies the study as qualitative and describes its focus on cardiac recovery following acute myocardial infarction from a gender perspective. | 1 |
|  | **Abstract** - The abstract summarises the study aim, qualitative design, methods, data sources, main findings and conclusion, including the gender-related patterns identified during cardiac recovery and their implications for nurse-led cardiac rehabilitation. | 1-2 |
|  |  |  |
| **Introduction** | |  |
|  | **Problem formulation** -The Introduction and Background describe the significance of cardiovascular disease and sex- and gender-related inequalities in cardiovascular risk, diagnosis and treatment. They identify the limited knowledge of how people experience the behavioural and psychosocial changes required during recovery after myocardial infarction and justify exploring these experiences from a gender perspective, including their relevance to nursing follow-up and support. | 3-4 |
|  | **Purpose or research question** -The study aims to explore, including from the gender perspective, how people who have suffered a myocardial infarction describe the changes they need to adopt during recovery following hospital discharge, in order to identify needs that may inform person-centred nursing assessment, follow-up and support. | 5 |
|  |  |  |
| **Methods** | |  |
|  | **Qualitative approach and research paradigm** - The study adopts a qualitative interpretative phenomenological approach. The theoretical framework is interpretative phenomenology, which is used to understand participants’ subjective experiences and recovery narratives. | 5 |
|  | **Researcher characteristics and reflexivity** - A member of the research team affiliated with the participating primary care centres contacted potentially eligible participants to invite them to participate. The study objectives were explained during the call. If the participant agreed to be interviewed, another member of the research team subsequently contacted them to arrange the location and date of the interview. Two researchers with experience in qualitative research conducted all interviews. Their experience and relationship with participants are described in the manuscript. | 6-8 |
|  | **Context** - The study was conducted in two primary healthcare centres located in an urban area in Spain, where participants were undergoing post-infarction follow-up. | 5 |
|  | **Sampling strategy** - A purposive theoretical sampling strategy with maximum variation was used to diversify discursive positions. Sample profiles were defined by gender, age, employment status and socioeconomic situation. Recruitment continued until data saturation was reached. | 5-6 |
|  | **Ethical issues pertaining to human subjects** - Ethical approval was obtained from the relevant Research Ethics Committees; the full names of the committees and approval/reference numbers are provided on the title page. All participants provided written informed consent before the interview. Participants were assigned alphanumeric codes, direct personal identifiers were removed from the transcripts, and confidentiality was protected throughout the research process. | 7–8; title page |
|  | **Data collection methods** - Data were collected through semi-structured interviews conducted between March 2022 and December 2023. Interviews were conducted in the language of the participant’s choice, either at the participant’s home or in a designated private room and lasted approximately one hour. All interviews were audio-recorded and transcribed verbatim, capturing verbal content and relevant non-verbal cues. | 6-7 |
|  | **Data collection instruments and technologies** - Semi-structured interviews were used to explore participants’ lived experience of cardiac recovery after hospital discharge, with attention to perceived lifestyle changes, emotional responses, social and family roles, work readaptation, sexuality and follow-up by the healthcare team. Interviews were audio-recorded and transcribed verbatim. | 6-7 |
|  | **Units of study** - The study included nine participants. Four participants were women and five were men. No participant identified as non-binary. Ages ranged from 44 to 78 years. Relevant participant characteristics are presented in Table 2. | 8-9; Table 2 |
|  | **Data processing** - The interview transcripts constituted the textual corpus for analysis. Data were analysed using thematic analysis combining deductive and inductive coding. Software-aided coding was conducted using ATLAS.ti. Participants were assigned alphanumeric codes, and the data were pseudonymised rather than fully anonymised because age, gender and other contextual characteristics were retained for analytical purposes. | 7-8 |
|  | **Data analysis** - The analytical procedure included repeated reading of transcripts, identification of meaningful text segments, software-aided coding, review of internal consistency of codes, categories and subcategories, review of consistency across categories, and development of results through code and category maps, explanatory texts and illustrative verbatim quotations. Themes and subthemes were refined through constant comparison and discussion among the research team until consensus was reached. | 7 |
|  | **Techniques to enhance trustworthiness** - Trustworthiness was addressed using the criteria of credibility, transferability, confirmability and dependability. Credibility was strengthened through participant validation of interview transcripts and reflexive dialogue among researchers. Transferability was supported by purposive sampling with maximum variation and contextual descriptions. Confirmability was enhanced through verbatim quotations and external peer review of the coding framework by a researcher with expertise in gender perspective. Dependability was supported by documenting analytical decisions and involving researchers who had not participated in data collection. The SRQR checklist guided the reporting of the study. | 7-8 |
|  |  |  |
| **Results/findings** | |  |
|  | **Synthesis and interpretation** - The main findings are presented in three themes: initiation of one’s own resources, engagement with one’s own emotions, and follow-up by the healthcare team. These themes describe participants’ experiences during cardiac recovery and include gender-related patterns concerning physical activity, return to work, household responsibilities and perceived continuity of care. A summary of themes and subthemes is provided in Table 3 | 9–16; Table 3 |
|  | **Links to empirical data –** Findings are supported with direct quotations from participants throughout the results section. The quotations illustrate experiences related to self-care, physical activity, comorbidities, return to work, household management, sexuality, family support, guilt, fear, discharge recommendations and post-discharge follow-up. | 10-16 |
|  |  |  |
| **Discussion** | |  |
|  | **Integration with prior work, implications, transferability, and contribution(s) to the field -** Findings are integrated with previous research on lifestyle change after myocardial infarction, adherence to cardiac rehabilitation recommendations, gender-related inequalities, return to work, household roles, sexuality, family support, psychological needs, continuity of care, nurse-led interventions and mHealth. The discussion addresses implications for primary care nursing, gender-sensitive cardiac recovery and nurse-led cardiac rehabilitation. Transferability is considered in relation to the sample size and socioeconomic context of the study area. | 16-24 |
|  | **Limitations** - The limitations include the small sample size, the socioeconomic context of the study area, the absence of participants identifying as non-binary, and the possibility of recall bias. The limited participation of interviewees in cardiac rehabilitation programmes is also discussed. | 22-23 |
|  |  |  |
| **Other** | |  |
|  | **Conflicts of interest** - The authors declare no conflict of interest. | Title page |
|  | **Funding** - Funding details are provided on the title page. | Title page |
|  |  |  |
|  |  |  |
|  | *The authors created the SRQR by searching the literature to identify guidelines, reporting standards, and critical appraisal criteria for qualitative research; reviewing the reference lists of retrieved sources; and contacting experts to gain feedback. The SRQR aims to improve the transparency of all aspects of qualitative research by providing clear standards for reporting qualitative research. |  |
|  |  |  |
|  | **The rationale should briefly discuss the justification for choosing that theory, approach, method, or technique rather than other options available, the assumptions and limitations implicit in those choices, and how those choices influence study conclusions and transferability. As appropriate, the rationale for several items might be discussed together. |  |
|  |  |  |
|  | **Reference:** |  |
|  | O'Brien BC, Harris IB, Beckman TJ, Reed DA, Cook DA. **Standards for reporting qualitative research: a synthesis of recommendations.** *Academic Medicine*, Vol. 89, No. 9 / Sept 2014  DOI: 10.1097/ACM.0000000000000388 |  |
|  |  |  |
|  |  |  |
